# Application of the Box–Behnken design for the production of soluble curcumin: Skimmed milk powder inclusion complex for improving the treatment of colorectal cancer

**DOI:** 10.1002/fsn3.1957

**Published:** 2020-10-30

**Authors:** Muthu Mohamed Jamal Moideen, Ali Alqahtani, Krishnaraju Venkatesan, Fazil Ahmad, Kalpana Krisharaju, Mohammed Gayasuddin, Rasheed Ahemad Shaik, Khalid Mohamad Morsy Ibraheem, Mohamed EL‐dosoky Mohamed Salama, Sally Yussef Abed

**Affiliations:** ^1^ Department of Pharmaceutical Technology BIT campus Anna University Tiruchirappalli India; ^2^ Department of Pharmacology College of Pharmacy King Khalid University Abha Saudi Arabia; ^3^ Department of Anesthesia Technology College of Applied Medical Sciences in Jubail Imam Abdulrahman Bin Faisal University Jubail Saudi Arabia; ^4^ Department of Pharmaceutical Analysis Erode College of Pharmacy Erode India; ^5^ College of Applied Medical Sciences King Saud bin Abdulaziz University for Health Sciences Al‐Ahsa Saudi Arabia; ^6^ King Abdullah International Medical Research Center Al‐Ahsa Saudi Arabia; ^7^ Department of Pharmacology & Toxicology Faculty of Pharmacy King Abdulaziz University Jeddah Saudi Arabia; ^8^ Department of Neuroscience Technology College of Applied Medical Science in Jubail Imam Abdulrahman Bin Faisal University Jubail Saudi Arabia; ^9^ Department of Respiratory Care College of Applied Medical Science in Jubail Imam Abdulrahman Bin Faisal University Jubail Saudi Arabia

**Keywords:** Box–Behnken design, colorectal cancer, dyeing test, inclusion complex, skimmed milk powder, thermodynamic parameters

## Abstract

The main objective of this study was to develop a soluble product of the practically insoluble curcumin (CMN) to treat colorectal cancer more effectively than with pure CMN. To improve the solubility of CMN, various hydrophilic carriers of skimmed milk powder (SMP), polyvinylpyrrolidone (PVP), and mannitol (MNT) were utilized to prepare solid dispersion (SD) binary complexes. The prepared complexes were characterized in terms of their aqueous solubility and in vitro drug release and analyzed by Fourier transform infrared spectrophotometry, powder X‐ray diffractometry, scanning electron microscopy, dynamic light scattering, and the novel dyeing test. Based on this characterization, the best SD complex was optimized using the Box–Behnken design (RSM‐BBD). These results showed that the solubility of CMN was greatly improved in combination with SMP. The SD of CMN with SMP produced significantly improved solubility (0.646 ± 0.024 mg/ml) and dissolution (54.94 ± 3.21% at 5 min). Further, solid‐state characterization revealed that the complex exhibited intermolecular inclusion of the drug and carrier. Also, the complex did not undergo any chemical modification owing to its amorphous form, and the novel dye test showed better coloring impact, indicating the solubility of CMN. The *in vitro* cytotoxicity of the complex showed that 50% inhibition (IC_50_) of SW480 and Caco‐2 cells was achieved at a considerably lower concentration than that of pure CMN. Flow cytometry analysis confirmed that the cell cycle arrest was at G2/M phase (43.26% and 65.14%), and DNA fragmentation analysis investigation confirmed that the complex induced more DNA damage during apoptosis.

## Graphical abstract

Developing a soluble product of the practically insoluble curcumin (CMN) to treat colorectal cancer more effectively than with pure CMN. Skimmed milk powder (SMP) used a solubility as well as cytotoxicity enhancer. Soluble CMN tremendously enhanced the solubility and cytotoxicity on human adenocarcinoma cells.

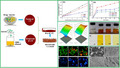

## 1 INTRODUCTION

Skimmed milk powder (SMP) is prepared from pure milk by pasteurization followed by evaporation, and then, the powdered soluble SMP can be obtained using spray dryer. The composition is fat (1%), protein (35%), carbohydrates (51%), minerals (7%), and moisture (3.5%) (Table 1). SMP is composed of alpha‐, beta‐, and kappa‐casein and acts as an emulsifying agent for oral pharmaceutical preparations (Shah et al., 2009). SMP can enhance drug solubility due to the hydrophobic protein component, that is, casein being entrapped in to a lipophilic/hydrophobic drug, and the reaction (inclusion complex) is caused by β‐casein acting as a surfactant. The drug is subsequently released due to the presence of kappa‐casein on the surface of the hydrophilic inclusion complex; this improves the wettability and surface tension of the complex, leading to enhancement of solubility of the drug (Palanisamy & Khanam, 2010).

**TABLE 1:** Protein and amino acids composition of SMP

Protein and protein fraction	App. % of skim milk protein	Essential amino acids	Content in gm/100 gm
1. Casein	79.5	Isoleucine	2.19
αs_1_‐Casein	30.6	Leucine	3.54
αs_2_‐Casein	8.0	Valine	2.42
β‐Casein	28.4	Methionine	0.91
k‐Casein	10.1	Phenylalanine	1.75
Casein fraction		Threonine	1.63
*Y*1, *Y*2, *Y*3—casein (from β‐casein)	2.4	Tryptophan	0.51
2. Whey protein		Lysine	2.87
(noncasein)	20.3	Histidine	0.98
β‐Lactoglobulin	9.8	–	–
α‐Lactalbumin	3.7	–	–
Blood serum albumin	1.2	–	–
Immunoglobulin	2.1	–	–
Miscellaneous	2.4	–	–

Most anticancer drugs can develop toxicity in the body. Synthetic or semisynthetic drugs may have various side effects such as bone marrow depression, alopecia, extreme fatigue, loss of self‐confidence, loss of immunity, and a remarkable decline in WBC count. The insolubility of anticancer drugs leads to their accumulation at the absorption site, and this may develop toxicity (Nurgali et al., 2018). The dosing frequency can be minimized by establishing the proper dosage form to deliver the drug over an extended period.

Numerous studies on curcumin (CMN) have proven that its principal active component—lipophilic polyphenol—produces various anticancer activity; however, its weak pharmacokinetics, including low solubility and stability as well as rapid metabolism and elimination, have limited its therapeutic application (Sachin et al., 2016).

The enhancement of the solubility of insoluble drugs is an important and one of the most challenging tasks in present‐day research. The diverse physicochemical methodologies that have been explored to enhance drug solubility include the addition of surfactants, nanosizing and micronization, conversion to the amorphous state, increasing wettability, developing prodrugs and salts, the liposome approach (Bansal et al., 2011), and SD. The basic mechanism by which SD improves the solubility of insoluble powder is by blending a carrier and drug, thus completely altering the state from semisolid/liquid to solid. Finally, the resultant product is milled to the greatest extent possible and then suitably sieved (Seo et al., 2012).

The Box–Behnken design (BBD) is an efficient choice of response surface methodology (RSM; Otto, 1999). Each factor has three levels, that is, low, center, and high. It is rotatable per factor with these three levels and joins a fractional factorial with inadequate block plans to avoid high peaks. This design is strictly limited to conditions where the researcher is not concerned with finding the response at the angles of the cube (boundaries). BBD is commonly used than the Doehlert design because it requires fewer number of experiments (Hanrahan et al., 2005). It requires greater skill and is ultimately analyzed by separating the number of coefficients to the quadratic condition by the number of analyses necessary for the design.

In this study, increasing the solubility of CMN was explored by preparing complexes with solid dispersion (SD) carriers of SMP, polyvinylpyrrolidone (PVP), and mannitol (MNT).

## 2 EXPERIMENTAL

### 2.1 Materials

Curcumin (purity >99%; SRL Pvt. Ltd), mannitol (S.D. Fine Chem. Pvt. Ltd), polyvinylpyrrolidone (PVP; Sigma‐Aldrich), and SMP (Aavin, Tamilnadu co‐operative milk product producers) were purchased. The colorectal adenocarcinoma cell lines of SW480 and Caco‐2 were obtained from the NCCS (National Center for Cell Science) in Pune. The cells were cultured in Dulbecco's modified Eagle's medium (DMEM; Sigma‐Aldrich) supplemented with fetal bovine serum (10%) and penicillin/streptomycin (1%, 20 ml; Hi‐media) as an antibiotic, at 37°C in a humidified environment of 5% carbon dioxide in a carbon dioxide incubator (Thermo Scientific). Other chemicals and reagents used were analytical grade.

### 2.2 Phase solubility study

The phase solubility (PS) study was according to the methodology of Higuchi and Connors (1965). Briefly, an excess amount of aqueous solution (25 ml) of different CMN concentrations (1%–15%) was added to the carriers (PVP, MNT, and SMP). The Eppendorf tubes containing this mixture were placed in a water bath at a steady temperature of 25 or 37°C ± 1°C for 24 hr to ensure that equilibrium was reached and were shaken at 30‐min intervals. The mixtures were then filtered through a Millipore membrane filter (0.45 µm), the filtrate diluted appropriately, and the absorbance measured at 425 nm by UV spectrophotometer. The stability complexation constant (*K*_1:1_) was ascertained (Equation 1) by using the slope and intercept of the solubility curve, with the intercept being directly proportional to the intrinsic solubility of CMN (Mohamed et al., 2019).

(1) $$K_{1:1}=\frac{S}{I(1‐S)}$$

Here, symbols *S* and *I* denote the slope and intercept, respectively. Additionally, the enthalpy change (ΔH) on complexation was obtained from the Van't Hoff equation (Equation 2).

(2) $$\ln\left(\frac{K_{2}}{K_{1}}\right)=\Delta H\frac{t_{2}‐t_{1}}{Rt_{2}t_{1}}$$

Here, *K*_2_, *K*_1_, and *t*_2_ and *t*_1_ refer to the stability constants and corresponding temperatures in Kelvin at 37 and 25°C, respectively. The Gibbs free energy change (Δ*G*) and change in entropy (ΔS) upon ideal complexation were computed from Equations 3 and 4, respectively.

(3) ΔG=‐RtlnK

Here, *R* is the universal gas constant (8.314 J mol^−1^ K^−1^).

(4) $$\Delta S=\frac{\Delta H‐\Delta G}{\Delta T}$$

### 2.3 Preparation of PM and SD

Physical mixtures (PM) of varying compositions (1:3 − 1:7 of CMN to carrier, respectively) were prepared with a mortar and pestle as specified by the guidelines of geometrical mixing and by sieving (# 120; 150 − 125 µm). SD complexes of same compositions were prepared by the following methods. 100 mg of CMN was used in each preparation.

#### 2.3.1 Solvent evaporation method

CMN was dissolved in ethanol, followed by addition of carrier (SMP/PVP). The resulting dispersion was stirred at 40 ± 0.5°C by magnetic stirrer (REMI‐2MLH) and then transferred to a petri dish heated to 50 ± 0.5°C on a hot plate to effect evaporation of the solvent. The obtained product was desiccated at 40°C for 48 hr (Verma et al., 2017).

#### 2.3.2 Melting method

The SD was prepared by adding CMN (100 mg) to the liquefied carrier (MNT) at 70°C with constant stirring at 700 rpm for 15 min until the product obtained as homogenous. The resulting product was allowed to solidify by cooling at 28°C, after which it was powdered, sieved, and stored in a desiccator (Vasoya et al., 2019).

### 2.4 Aqueous solubility study

An excess quantity of sample (pure CMN, SD, or PM) was added to Milli Q water in a 100‐ml volumetric flask and heated in a water bath at a steady temperature of 37 ± 1°C for 24 hr, with shaking at 30‐min intervals (Mohamed et al., 2019). Subsequently, the mixture was filtered through a 0.45‐µm Millipore membrane filter and diluted to a suitable concentration and the UV absorbance was measured.

### 2.5 Dyeing experiment

A simple novel dye test can be applied to determine the effective solubility of a sample (usually colored) in aqueous media (Lanxiang et al., 2017). Briefly, CMN (10 mg) and its SDs (equiv. wt.) were added to double distilled water (15 ml). The resulting mixture was sonicated for 5 min followed by filtration, and then, images of the solutions were taken. The above solution was then diluted to 50 ml, and six white linen cloths (8 × 4.5 cm^2^) were soaked in it for 1.5 hr. The cloths were then dried at room temperature and observed for color intensity.

### 2.6 Solid‐state characterization

The individual carriers, CMN, drug–polymer PMs, and SDs were subjected to instrumental analysis to characterize their solid‐state properties. The Fourier transform infrared spectrophotometry (FT‐IR) and powder X‐ray diffractometry (PXRD) analyses were performed as described in our previous research (Moideen et al., 2019).

### 2.7 Dissolution study

The dissolution study was performed in 900 ml of double distilled water at 37 ± 1°C at 50 rpm using a USP dissolution apparatus II (DS 8000, Lab India). The sample was placed into the jar and the time set as zero. At each 5‐min interval until 30 min, 5 ml sample aliquots were syringed out and filtered through using 11‐µm Whatman filter paper. The percentage of dissolved CMN was estimated by UV spectrometer. A correction for the collective dilution initiated by addition of the sample to the double distilled water was applied to maintain the sink condition.

### 2.8 Optimization by BBD

The BBD is a dynamic second‐order trial configuration related to the minimum trial experiments. BBD was employed for optimization of the SD complex preparation variables. The Design‐Expert® version 12 software trial (Stat‐Ease Inc.) was utilized for arithmetical exploration by ANOVA, enabling construction of model equations and 3D response plots for each outcome. Carrier concentration (*X*_1_), stirring rate (*X*_2_), and temperature (*X*_3_), and the corresponding dependent variables (*Y*_1_ and *Y*_2_), aqueous solubility (Sol_aq_, mg/mL), and release in 5 min (Rel_5 min_, %) are the factors that facilitate the preparation of powder complexes (Chaudhary et al., 2011). The quadratic RSM was fitted to the following Equation 5. In each preparation, 100 mg of CMN was used.

(5) $$Y=\beta_{0}+\beta X_{1}+\beta_{2}X_{2}+\beta_{3}X_{3}+\beta_{12}X_{1}X_{2}+\beta_{13}X_{1}X_{3}+\beta_{23}X_{1}X_{3}+\beta_{11}X_{1}^{2}+\beta_{22}X_{2}^{2}+\beta_{33}X_{3}^{2}$$

### 2.9 Dynamic light scattering analysis (DLS)

The hydrodynamic particle size (PS), polydispersible index (PDI), and zeta potential (ZP) of the PM and SD complexes were determined by DLS (Mohamed et al., 2019) using a Zetasizer Nano ZS90 (Malvern Instruments Ltd.).

### 2.10 Scanning electron microscopy (*SEM*)

The surface characteristics of samples were examined using SEM to observe electrons moving in zig‐zag patterns (Carl Zeiss Microscopy Ltd, EVO 18). The test samples were diluted with Milli Q water (1:100), dropped onto two‐sided carbon adhesive tape pre‐affixed on a sample stub, and then allowed to dry at 28°C (Kumar et al., 2017). A thin layer of sample was gold coated (100 Å) by a sputter coater, following which the samples were observed at 5.0 kV.

### 2.11 MTT assay

The cytotoxicity of complexes against Caco‐2 and SW480 cell lines was estimated by MTT assay (Dhananjay et al., 2014). Briefly, cells were seeded at a density of 5 × 10^3^ cells·mL^−1^ (200 µl/well) in 96‐well culture plates with DMSO as the solvent. After 24 hr of incubation with different concentrations of complex, MTT reagent (5 mg/ml, 20 µl/well) was added to the medium and incubated at 37°C for 4 hr. The obtained purple formazan precipitate was solubilized by addition of DMSO (100 μl) to each well. The cell passage number was 3. The absorbance of each well was estimated at 570 nm by a plate reader (Bio‐Rad, iMark). Experiments were performed in triplicate, and the mean calculated. The IC_50_ value was determined as the concentration of complex required to decrease the absorbance to half the percentage of the control (Lakshmipraba et al., 2013).

### 2.12 Apoptosis Study

#### 2.12.1 Acridine orange (AO) and ethidium bromide (EB) staining

The AO/EB dual stain method was used to investigate the apoptotic morphology, with some modifications. Briefly, the IC_50_ concentration of compounds was used to treat the cells for 24 hr, which were then washed with cold PBS. Cells were then resuspended in PBS at a concentration of 5 × 10^5^ cells/ml and then mixed with 25 µl of AO/EB solution (3.8 µM of AO and 2.5 µM of EB in PBS) on a clean slide (Rajiu et al., 2015). These samples were quickly analyzed by fluorescence microscopy (Carl Zeiss, Axioscope 2plus) with a UV filter (450−490 nm). For each sample, 300 cells were counted for the number of live, necrotic, or apoptotic cells by staining the nucleus assembly and membrane integrity, and the percentage was calculated (Ramachandran et al., 2019). Morphological variations were also observed and micrographed (400× magnification).

#### 2.12.2 Hoechst staining

The Caco‐2 and SW480 cells were seeded in separate 6‐well plates and treated using the IC_50_ concentrations. After incubation for 24 hr, cells were collected and stained by aqueous Hoechst 33258 stain at room temperature for 5 min (Nagaraj et al., 2014). Morphological variations were also observed and micrographed by fluorescence microscopy with a 377−355 nm filter (400× magnification).

### 2.13 DNA fragmentation assay

DNA fragmentation was performed using the agarose gel electrophoresis method. The SW480 and Caco‐2 cells were cultured at 1 × 10^6^ cells/ml in 6‐well culture plates. Nearly 70% of the cells joined together, and the inclusion complex was added and incubated for 48 hr. After incubation, cells were collected and the pellets centrifuged (Eppendorf centrifuge 5430R) and washed twice with ice‐cold PBS. The pellets were lysed by treating with EDTA (10 mM), Tris‐HCl (10 mM), and Triton X‐100 (0.2%; pH 7.5) in cold buffer for 10 min. The mixtures were then centrifuged at 4°C and 13,000 *g* for 10 min, and the supernatant containing fragmented DNA, RNA, and undamaged chromatin was treated with IA‐P‐C (1:24:25) (Dasiram et al., 2017). The pellet was washed with ethanol (70%), dried at room temperature, and dissolved in T10E1 buffer (adjusted to pH 7.4, 20 μL). Agarose (2%) gel electrophoresis was utilized to assay 0.6% mg/mL of RNase A at 37°C for 30 min. At the end point, ethidium bromide was used to stain the gels and the DNA fragment observed by UV transillumination.

### 2.14 Flow cytometry

Caco‐2 and SW480 cells at 1 × 10^5^ cells/mL were incubated with the IC_50_ concentration as determined above for 24 hr to analyze the effect of the drug on the cell cycle distribution. The treated cells were collected and washed with phosphate buffer (pH 7.4), then treated with ethanol (80%) at 4°C overnight, and then washed twice with cold PBS. The washed cells were treated with propidium iodide stain (40 μg/ml) and RNase A (100 µg/ml) in PBS and shaken at 37°C for 30 min (Mosieniak et al., 2012). The cells were studied by flow cytometry (Becton Dickinson FACScan), and the % of cells in different phases was analyzed using Win MDI 2.9 (TSRI).

## 3 RESULTS AND DISCUSSION

### 3.1 Outcomes of PS study

The PS study confirmed the proposed effect of the various carriers on improving the solubility of CMN. A standard linear curve was obtained in the concentration ranges from 5.97 × 10^−5^ to 2.04 × 10^−4^ mM and 8.14 × 10^−5^ to 2.12 × 10^−4^ mM for CMN at 25 and 37°C, respectively (Table 2).

**TABLE 2:** Thermodynamic parameters of CMN with various carriers (mean ± *SD*, *n* = 3)

Carrier	T (°C)	Intercept (mM)	Slope (M)	Ka (M^−1^)	ΔG (kJ/mol)	ΔH (kJ/mol)	ΔS (kJ/molK)
SMP	25	2.04 * 10^–4^	0.01998	100.14 ± 6.17	−11.41 ± 0.215	7.20 ± 0.875	0.06245 ± 0.006
	37	2.12 * 10^–4^	0.02317	112.05 ± 7.12	−12.16 ± 0.256		
PVP	25	1.38 * 10^–4^	0.12522	1,033.91 ± 34.07	−17.21 ± 0.312	44.8 ± 3.124	0.20812 ± 0.004
	37	1.46 * 10^–4^	0.23382	2081.81 ± 44.17	−19.72 ± 0.345		
MNT	25	5.97 * 10^–5^	0.00062	10.88 ± 1.44	−5.78 ± 0.076	7.35 ± 0.868	0.04399 ± 0.002
	37	8.14 * 10^–5^	0.00094	11.90 ± 1.59	−6.33 ± 0.089		

Note

At 25 and 37°C.

**FIGURE 1:**
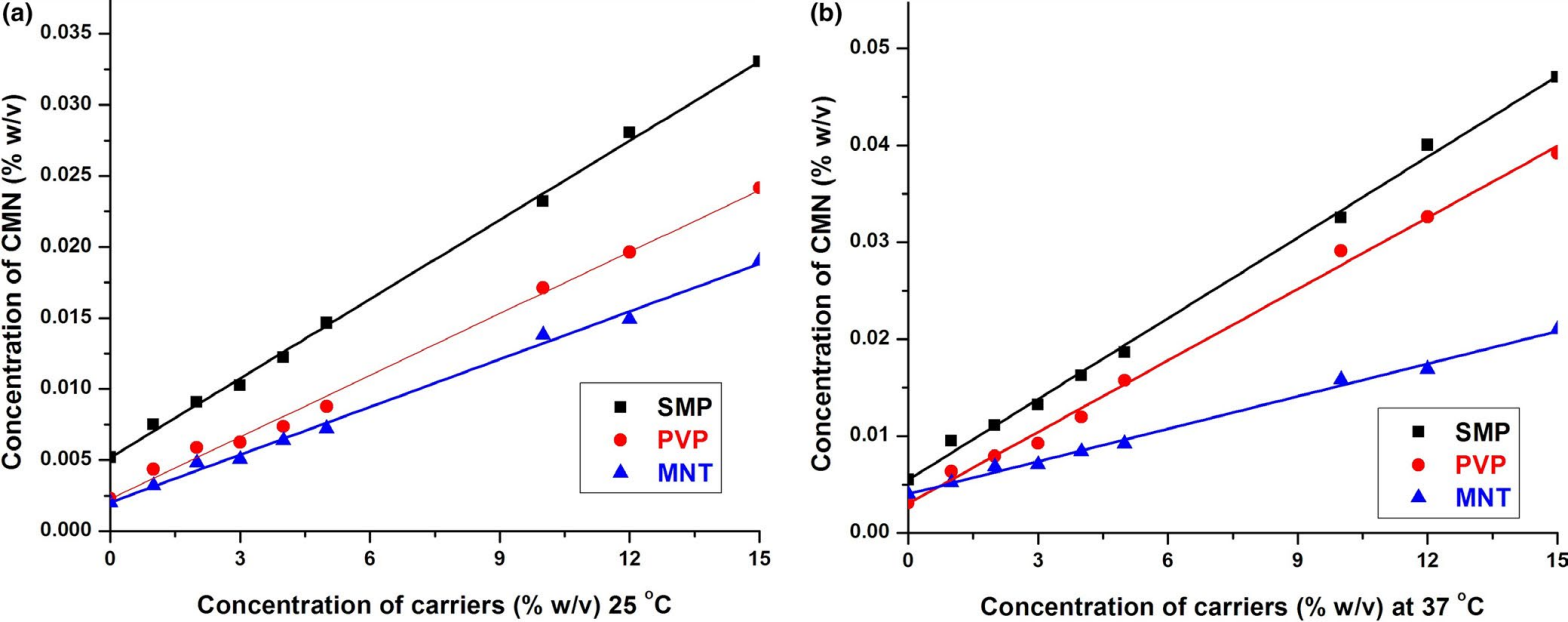
PS diagram of CMN in water (a) at 25 and (b) 37°C in the presence of SMP, PVP, and MNT

The solubility of CMN was enhanced linearly with increasing carrier concentration. With increasing concentration of carrier and temperature, the solubility of CMN increased progressively due to modification of interactions such as hydrophobic and Van der Waals forces between CMN and the carriers. The slope of the PS curve (usually <1) in all carriers revealed a 1:1 stoichiometry complex and A_L_‐type phase solubility profile (Bandari et al., 2014; Le et al., 2014). The apparent rate constant (*K*_1:1_) was calculated from the intrinsic intercept and slope value of solubility curves obtained by plotting the concentration of soluble CMN (% w/v) against the concentration of the carrier (% w/v) as shown in Figure 1.

The stability constant (Ka) of the complexes ranked in the following order, at 25 and 37°C, respectively: PVP (1,033.94 and 2,081.81 M^−1^) > SMP (100.14 and 112.05 M^−1^) > MNT (10.88 and 11.90 M^−1^). The entropy (Δ*S*), Gibbs free energy (∆*G*), and enthalpy (Δ*H*) were also determined, from the phase solubility diagram. Out of all the carriers, SMP had a perfect complexation constant within the range of 100 to 1,000 M^−1^; in contrast, MNT showed weak interaction with CMN and the interaction of PVP was too strong (PVP ≈ 1,033 and 2,081 M^−1^ at 25 and 37°C, respectively). Iacovino et al. (2012) demonstrated that a complex does not dissociate readily if its stability constant is greater than 1,000 M^−1^.

The determined values of Δ*G* were negative for all carriers, thus confirming the spontaneity of binding. Additionally, Δ*G* decreased with increasing molecular weight of the carrier. The determined values of Δ*H* were found to be positive (endothermic) for all carrier complexes, which was confirmed by the high values of ΔS for all carrier systems (6, 20 and 6 J mol^−1^ K^−1^).

The solubility of the CMN‐SMP inclusion complex with very low −Δ*S* value (0.5 J mol^−1^ K^−1^) was obtained because the ligand molecules were not ionized, and the water molecules were less ordered. The surface active properties and amino acid content of SMP (protein; casein)—which acts as an emulsifier and surface active agent—may lead to strong interaction with CMN as the hydrophobic hydrogen groups (Hartel & Hasenhuettl, 2008). Therefore, this result was obtained due to the establishment of a soluble complex between the polymeric carrier and lipophilic drug.

The CMN‐PVP complex was practically unsuitable because of its narrow solubility characteristics. Hydrophobic interactions between the hydrogen bond of CMN with the carbonyl group of PVP were likely involved between CMN and PVP (Chatjigakis et al., 1992). This interaction may have impacted the entropy, with water molecules accumulating around exposed nonpolar solute particles; however, when two nonpolar molecules approached, the disorder of water molecules increased, causing a positive change to the entropy and free energy of association.

An exception was noticed for MNT where a high + Δ*S* value (4.4 J mol^−1^ K^−1^) was obtained due to ionization of ligand molecules, resulting in water molecules being less ordered. The binding process was endothermic (+Δ*H*); Δ*H* favors Δ*G* and Δ*S*, and the spontaneity ensured by negative ΔG meant that at least complex formation occurred between MNT and CMN.

### 3.2 CMN solubility

The solubility of pure CMN in distilled water was found to be 0.004 mg/ml at 37°C after 24 hr. The solubility of CMN‐SMP (1:3 to 1:7) *SD* was enhanced ~120‐ to 150‐fold compared to that of pure CMN (Table 3). In contrast, the PVP and MNT binary systems showed less enhancement of solubility—25‐ to 30‐fold and 16‐ to 23‐fold, respectively—due to less interaction of these carriers with CMN (El‐Badry, 2011). The amino acid content of SMP was primarily responsible for the greater solubility enhancement of the CMN‐SMP complex.

**TABLE 3:** Aqueous solubility of CMN‐PM and SD complexes (mean ± SD, n = 3).

	Aqueous solubility (mg/ml)				
	Pure CMN at 37°C			0.004 ± 0.0003	
D:C ratio*	1:3	1:4	1:5	1:6	1:7
CMN‐PM					
SMP	0.2145 ± 0.077	0.2267 ± 0.087	0.2674 ± 0.065	0.2855 ± 0.066	0.2910 ± 0.072
MNT	0.0211 ± 0.034	0.0225 ± 0.041	0.0269 ± 0.041	0.0291 ± 0.063	0.0321 ± 0.044
PVP	0.0102 ± 0.047	0.0166 ± 0.057	0.0172 ± 0.073	0.0181 ± 0.078	0.0862 ± 0.076
CMN‐SD					
SMP	0.4872 ± 0.028	0.4919 ± 0.016	0.5288 ± 0.024	0.5525 ± 0.025	0.5791 ± 0.024
MNT	0.0671 ± 0.028	0.0698 ± 0.025	0.0795 ± 0.022	0.0812 ± 0.032	0.0912 ± 0.034
PVP	0.1012 ± 0.031	0.1056 ± 0.020	0.1132 ± 0.017	0.1189 ± 0.031	0.1212 ± 0.032

* Drug: carrier ratio

Overall, the aqueous solubility of CMN in the SDs was enhanced to a greater degree—depending on the carrier concentration—than in the PMs. The aqueous solubility of the PMs was of less importance than the solubility of the SDs due to the partial or improper complexation of the carriers with CMN.

### 3.3 Dyeing effect

**FIGURE 2:**
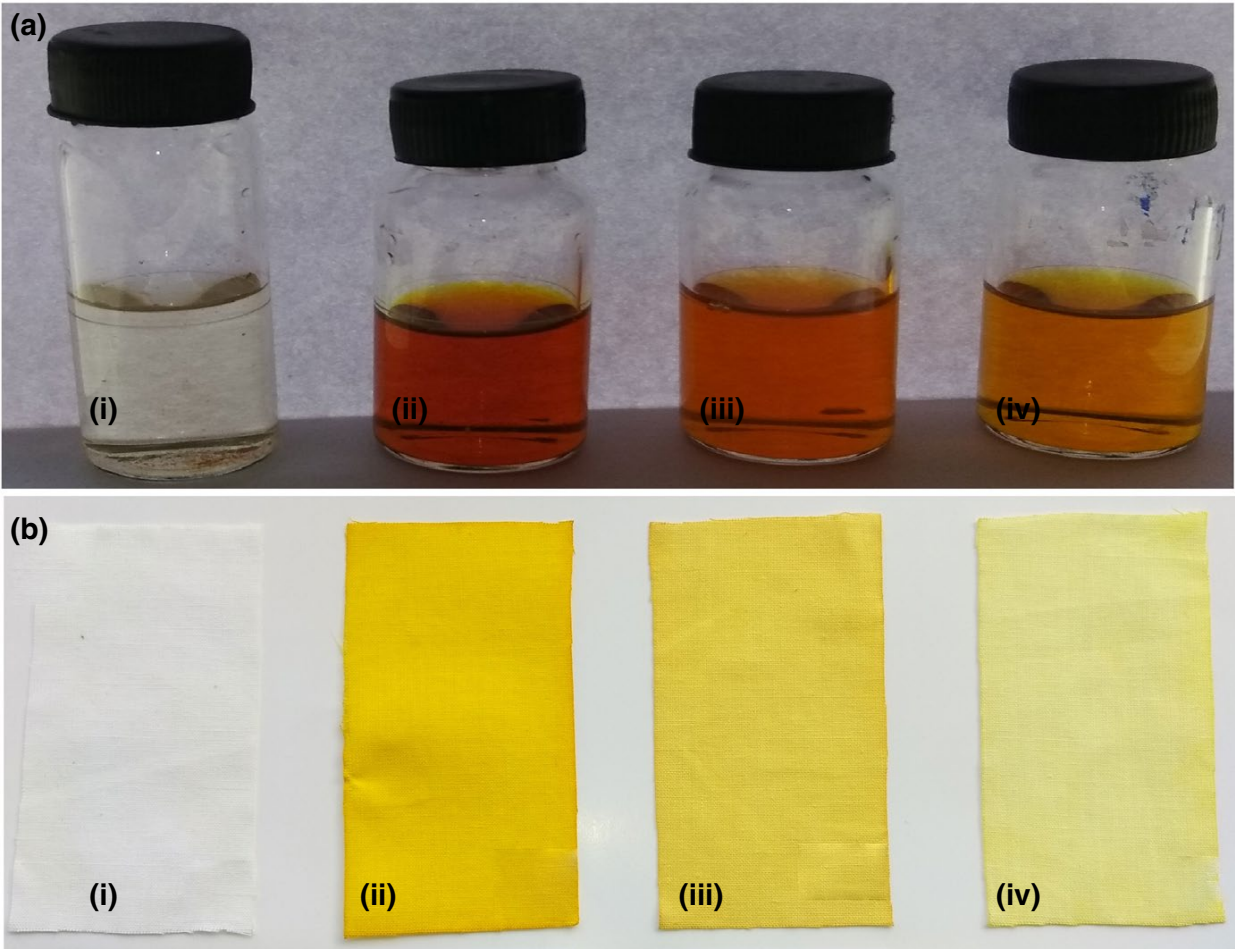
Photograph of the dye test (a) Solution of *SD* and (b) Cotton clothes dyed in the solution (i) Pure CMN, (ii) CMN‐SMP, (iii) CMN‐PVP, and (iv) CMN‐MNT

Results of the dyeing test for pure CMN and its complex SDs with SMP, PVP, and MNT are shown in Figure 2. When 10 mg CMN was added to 15 ml of water, CMN floated due to its lipophilic nature and the solution was colorless. For the SDs with SMP, PVP, and MNT, the solution was deep yellow, yellow, and pale yellow, respectively (Figure 2a). Figure 2b shows that the cloth stained in the solution was colorless for pure CMN, characteristic yellow (SMP), yellow (PVP), and pale yellow color (MNT).

The inference of these dying effects was that the solubilizing capacity was greatest for the CMN inclusion complex with SMP. This direct, novel dyeing test demonstrated that the CMN‐SMP SD had better coloring impact, demonstrating that the solubility of CMN in water increased the most after incorporation in this complex (Xu et al., 2015).

### 3.4 Physicochemical results

#### 3.4.1 FT‐IR

**FIGURE 3:**
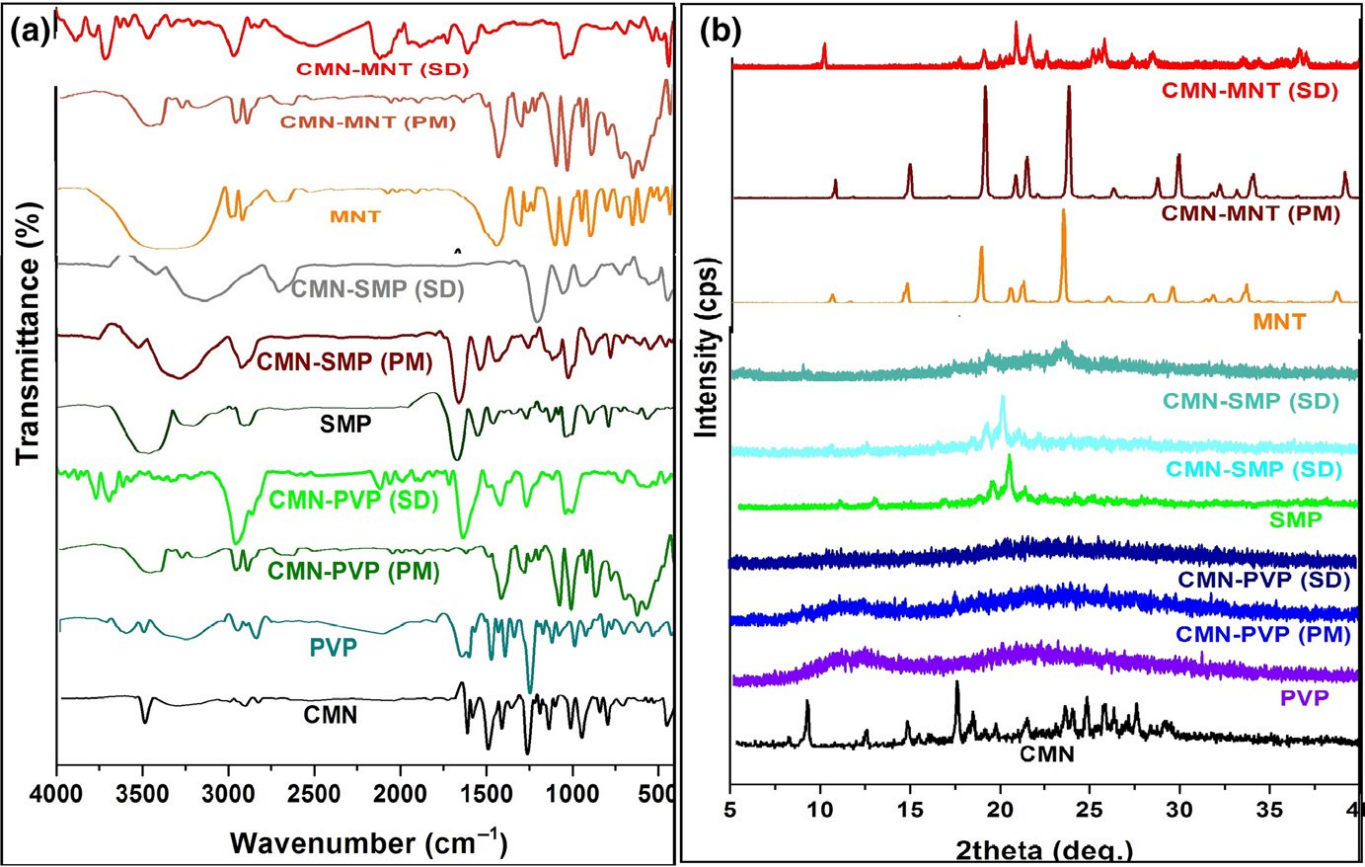
FT‐IR spectrum (a) and PXRD pattern (b) of pure CMN, carriers PM, and its *SD*

FT‐IR spectra of pure CMN, the carriers, and the PM and *SD* complexes are shown in Figure 3a. The CMN spectrum contained two peaks corresponding to ‐OH (alcohol) stretch vibrations at 3,324.68 and 3,015.16 cm^−1^ indicating the existence of two hydroxyl groups. The peaks at 1742.37 and 1629.55 cm^−1^ indicated the presence of C=O (carbonyl) groups in the structure (Kurniawansyah et al., 2017). The C−H (alkane) stretch vibrations at 2,922.59 and 2,849.31 cm^−1^ and C = C (aromatic) stretch vibrations at 1597.73 and 1507.1 cm^−1^ were also evident.

For the SD complex of CMN and SMP, the C=O stretching vibrations vanished, indicating that the interaction occurred with the carrier through hydrogen bonding. The SMP peak at 3,460.63 cm^−1^ and the alkene stretching vibration at 1662.34 cm^−1^ demonstrated the intermediate complex. Some peaks that were present in the CMN spectrum were also present in the SMP complex, with O−H and C−H stretching vibrations observed in the PM and *SD* spectra (Figure 3a). The disappearance of the peak corresponding to C=O in CMN was indicative that some interactions occurred. The characteristic peaks of MNT observed at 3,398.92, 2,969.84, 2,701.78, 2,513.76, 1744.3, and 962.30 cm^−1^ corresponded to O−H, C−H, O−H (carboxylic acid), S−H thiol, C−O stretching, and C−H bending vibrations, respectively. In the PM and SD of CMN‐MNT, there was a marked decrease in the intensity of OH stretching vibrations compared to in the CMN spectrum (Figure 3a). The C−H stretching vibration due to MNT was sustained both in the SD and PM. The greatly decreased intensity of the C=O peak of CMN in the complex indicated a hydrogen bonding interaction.

The characteristic peaks of PVP observed at 3,450.99, 2,922.59, 1662.34, and 934.34 cm^−1^ corresponded to O−H broadening, C−H expanding, C=C broadening, C−O broadening, and C−H bending vibrations (Fule et al., 2013). In the PM, the characteristics of the drug were evident and the significant single O−H broadening vibrations observed at 3,450, 2,922, and 1662.59 cm^−1^ in the SD showed that the communication between the O−H bond of the solution and carbonyl group of PVP had hardly been affected.

#### 3.4.2 PXRD

The PXRD patterns of CMN, the carriers, PMs, and SDs are shown in Figure 3b. CMN displayed sharp peaks (indicating a crystalline arrangement) at 2θ = 8.98º and 17.38º and many tiny peaks at 23.48º, 24.72º, 25.68º, 26.22º, and 27.5º (Jang et al., 2014). Many diffraction points with high intensity were observed for MNT, with strong and intense peaks at 2θ = 18.96, 21, and 23.54º. The same MNT X‐ray diffraction pattern (Figure 3b) was observed in the PM. In contrast, the CMN peak at 2θ = 9.28° was decreased in intensity in the SD; this was because there were no changes in crystallinity, and the peaks of MNT remained unchanged, indicating that there was little interaction occured between the drug and this carrier.

In contrast, PVP and SMP showed amorphous characteristics because of their lack of comprehensive stereo uniformity and exist the large lateral groups in these carrier (Figure 3b). The obtained X‐ray diffraction patterns revealed that this amorphous nature was fundamental in SMP‐PM because its low intensity was due to the low amount of CMN dissolved. In contrast, the huge amount of drug solubilized in the amorphous assembly of the solid carrier matrix was demonstrated by various characteristic peaks of the SD, signifying alterations to the crystalline lattice of CMN by the amorphous form of SD. Both the PMs and SDs of PVP and SMP displayed an indeterminate structure with decreased intensity at 2θ = 8.98° and 17.38° when compared to that of crystalline CMN.

### 3.5 CMN release state

**FIGURE 4:**
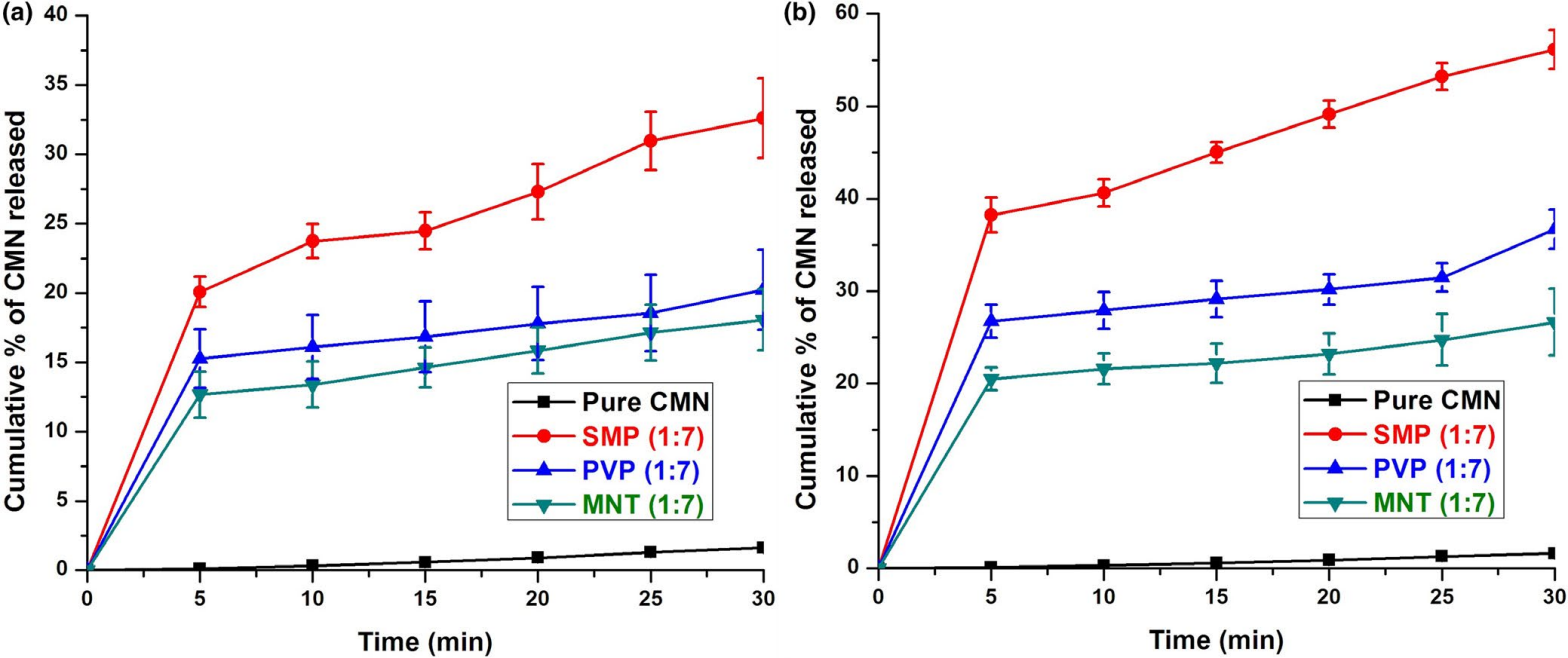
Cumulative drug release of CMN from (a) PM and (b) SD (mean ± *SD*, *n* = 3)

The average dissolution curves of CMN and its PM and SD are shown in Figure 4. It is notable that the dissolution rate of pure CMN was lowest (1.62%) at around 30 min due to its high hydrophobicity. The dissolution rate of the complexes of CMN and the carriers exhibited great burst release (12%–40%) in the initial 5−6 min (Table 4); this showed that the entire complex established with the carrier or was converted to an amorphous mass (Sadeghi et al., 2016).

**TABLE 4:** Cumulative percentage release of CMN

PM	Time	5	10	15	20	25	30
Pure CMN	0	0.11 ± 0.02	0.32 ± 0.04	0.58 ± 0.04	0.90 ± 0.06	1.35 ± 0.06	1.62 ± 0.07
SMP (1:7)	0	20.07 ± 1.09	23.74 ± 1.32	24.48 ± 1.33	27.3 ± 2.01	30.97 ± 2.15	32.61 ± 2.88
PVP (1:7)	0	15.26 ± 2.13	16.11 ± 2.37	16.84 ± 2.56	17.8 ± 2.65	18.55 ± 2.78	20.24 ± 2.88
MNT (1:7)	0	12.67 ± 1.68	13.39 ± 1.66	14.62 ± 1.71	15.85 ± 1.83	17.14 ± 2.03	18.06 ± 2.28
CMN‐*SD*							
SMP (1:7)	0	38.24 ± 2.88	40.63 ± 2.75	45.02 ± 2.78	49.14 ± 2.81	53.22 ± 2.99	56.15 ± 3.12
PVP (1:7)	0	26.74 ± 1.78	27.92 ± 1.97	29.15 ± 1.37	30.18 ± 1.65	31.47 ± 1.54	36.69 ± 2.11
MNT (1:7)	0	20.48 ± 1.32	21.58 ± 1.32	22.20 ± 1.32	23.20 ± 1.32	24.72 ± 1.32	26.63 ± 1.32

Note

Time in minutes.

Abbreviation: PM, physical mixture.

After 5 min, low quantities of CMN were released at a steady rate for all PMs and SDs. In SMP SD (1:7), 56.15% of the drug was released toward the end of 30 min with an initial burst release of 38.34% (Figure 4a), release of 20.07% at 5 min, and 32.64% at 30 min for the SMP‐PM (1:7) (Figure 4b). The enhanced dissolution of the drug in SMP was exhibited as due to the strong interaction taking place. The presence of amino acids and proteins in SMP increased the solubility of CMN by molecular dispersion of the SD complex (Palanisamy & Khanam, 2010). Dissolution of these carriers could be due to the metastable supersaturation of CMN in the concentrated wet carrier during dissolution (Li et al., 2015). Thus, SMP gave the best solubility up to a 1:7 ratio, at higher ratios of carrier the solubility declined, indicating that dissolution is dependent on carrier concentration. The in vitro release demonstrated that, after the burst release, a steady rate release profile was seen with all SDs (Table 4).

Rapid dissolution of CMN, slightly less than SD, may be clarified as an in situ complex improvement arising from decreased interfacial tension (Figure 4a). Remarkably, the hydrophilic carrier PVP increased the wettability and spreadability of CMN by lessening the amount of CMN that was immediately soluble. PVP is a hydrophilic carrier, and, the viscosity of the binary system increased, which in turn decreased the dispersion of the drug molecule and helped to form a crystal lattice. The CMN‐SD obtained with MNT was not satisfactory in comparison to the other carriers; this was likely due to the polar effect of the carbohydrates and the formation of hydrogen bonds improving solubilization (Saharan et al., 2009). This result showed that the limited dissolution rate of MNT could due to its partial conversion from the crystalline to the amorphous form in the presence of pure CMN powder. As an outcome of these findings, the low dissolution rate of MNT may be a result of inadequate complexation or amorphization of the CMN particles, compared with those of pure CMN.

### 3.6 Optimization of Variables

The experimental results showed that the CMN‐SD SMP binary system prepared by the solvent evaporation method had enhanced solubility and dissolution of CMN, with better physicochemical properties. Subsequently, BBD was employed for the optimization of the SMP SD complex. The carrier (SMP) concentration (*X*_1_), stirring rate (*X*_2_), and temperature (*X*_3_) were the independent variables for the three‐factor, two‐level design, and the dependent variables selected were aqueous solubility (Y_1_; Sol_aq_, mg/ml) and release in 5 min (Y_2_; Rel_5 min_; Table 5).

**TABLE 5:** Effect of independent process variables on dependent variable by Box–Behnken design

Preparation code	Real value (coded level)			Sol_aq_ (mg/ml)		Rel_5min_ (%)	
				Actual	Predicted	Actual	Predicted
	*X*_1_	*X*_2_	*X*_3_	*Y*_1_	*Y*_1_	*Y*_2_	*Y*_2_
P1	500 (−1.00)	500 (−1.00)	55 (0.00)	0.34	0.35	47.53	46.30
P2	1,000 (1.00)	500 (−1.00)	55 (0.00)	0.51	0.51	49.14	52.50
P3	500 (−1.00)	1,000 (1.00)	55 (0.00)	0.35	0.36	46.46	48.85
P4	1,000 (1.00)	1,000 (1.00)	55 (0.00)	0.59	0.58	48.95	53.18
P5	500 (−1.00)	750 (0.00)	40 (−1.00)	0.41	0.42	51.96	55.13
P6	1,000 (1.00)	750 (0.00)	40 (−1.00)	0.67	0.68	52.38	55.13
P7	500 (−1.00)	750 (0.00)	70 (1.00)	0.38	0.39	49.52	52.24
P8	1,000 (1.00)	750 (0.00)	70 (1.00)	0.62	0.61	53.54	55.13
P9	750 (0.00)	500 (−1.00)	40 (−1.00)	0.53	0.54	53.96	49.40
P10	750 (0.00)	1,000 (1.00)	40 (−1.00)	0.57	0.57	52.42	47.63
P11	750 (0.00)	500 (−1.00)	70 (1.00)	0.51	0.50	53.44	49.66
P12	750 (0.00)	1,000 (1.00)	70 (1.00)	0.51	0.51	52.39	53.85
P13	750 (0.00)	750 (0.00)	55 (0.00)	0.55	0.56	55.23	55.13
P14	750 (0.00)	750 (0.00)	55 (0.00)	0.54	0.56	54.75	53.72
P15	750 (0.00)	750 (0.00)	55 (0.00)	0.55	0.56	54.73	55.13
P16	750 (0.00)	750 (0.00)	55 (0.00)	0.55	0.56	55.21	51.98
P17	750 (0.00)	750 (0.00)	55 (0.00)	0.54	0.56	55.68	52.66

Note

X_1_: carrier concentration (mg); X_2_: stirring speed (rpm); and X_3_: temperature (°C) and Y_1_: aqueous solubility (mg/mL); and Y_2_: release at 5 min (%).

The independent coded values of variables were as follows. Carrier concentration (CC): low value (−1) 500 mg, high value (+1) 1,000 mg; stirring rate (SR): low value (−1) 500 rpm, high value (+1) 1,000 rpm (REMI‐2MLH); and temperature (Temp): low value (−1) 40, high value (+1) 70°C). These were selected based on the outcomes from earlier experimentation (Seelan et al., 2016): Seventeen experiments were performed with different conditions for the preparation of CMN‐SMP inclusion complex.

#### 3.6.1 Sol_aq_

The quadratic models were fitted for the dependent variables by the software. The polynomial model for a dependent variable Y_1_ is denoted by the following equation (Equation 6):

(6) $$\begin{array}{cc} Y_{1}= & +0.5460+0.13*X_{1}+0.014*X_{2}‐0.020*X_{3}‐0.010*X_{1}*X_{2}‐0.005*X_{1}*X_{3}‐0.0010*X_{2}*X_{3}‐\\ & 0.0010*X_{2}*X_{3}‐0.050*X_{1}^{2}‐0.041*X_{2}^{2}+0.025*X_{3}^{2}. \end{array}$$

The report for the analysis of variance (ANOVA) representing the quadratic predictive model was significant (*p* = .0001). The *R* ^2^ value .9986 for *Y*_1_ (Sol_aq_; mg/ml) showed the similarity of the predicted (0.9885) and experimental responses, and the values of CV (1.09) and adj.*R* ^2^ (.9968) indicated a high rate of precision and reliability of the investigated values and a high rate of correlation between the predicted and observed values, respectively. The other factors and their interactions were not significant (*p* > .05).

**FIGURE 5:**
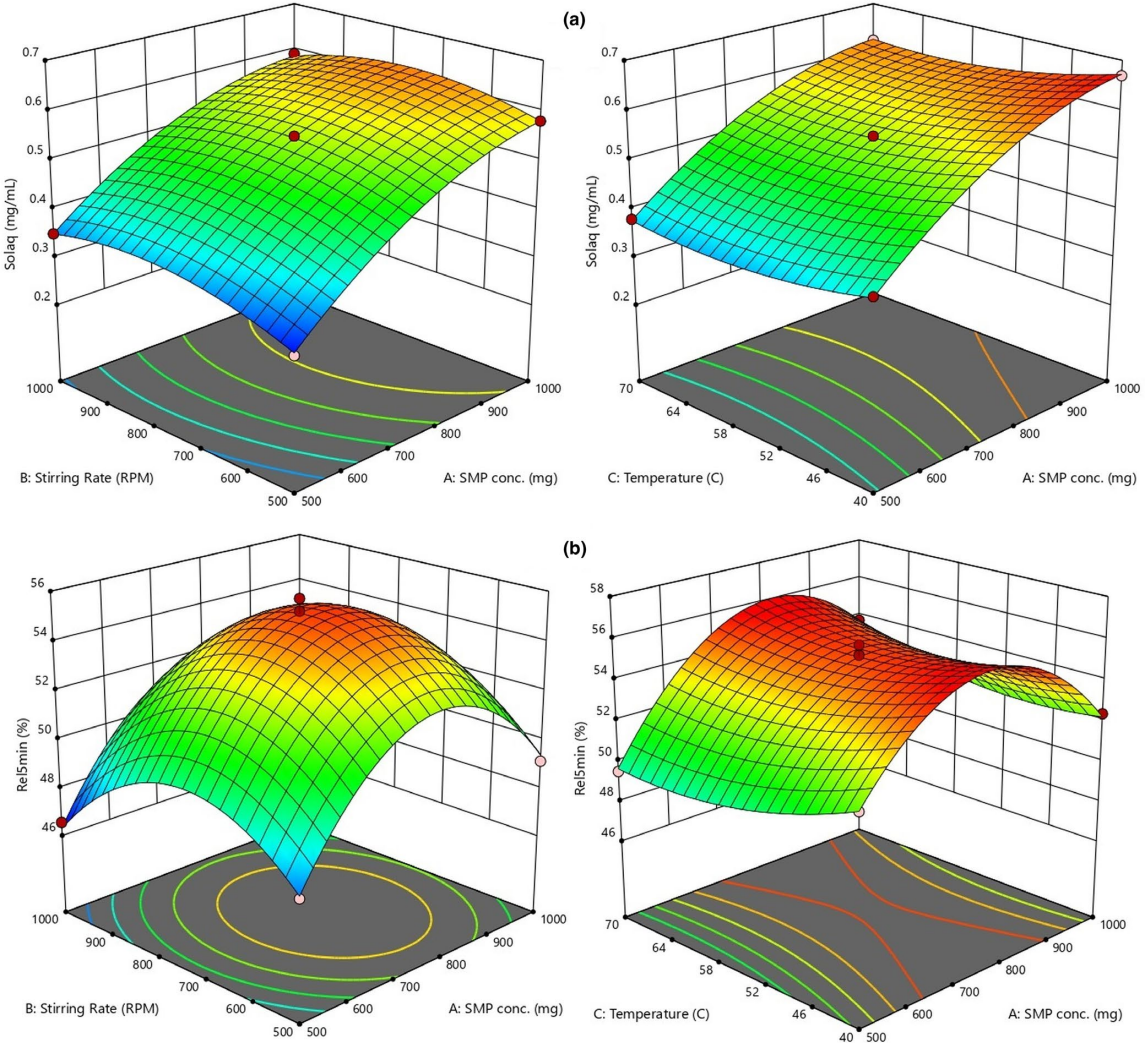
Response surface plots (3‐D) showing the effect of process variable on (a) aqueous solubility (Solaq; mg.mL‐1) and (b) release at 5min (Rel5min; %) from CMN‐SMP complex

The interaction properties of the dynamic response were validated by graphical representation, which were dependent on the CC (*X*_1_), SR (*X*_2_), and Temp (*X*_3_), and the resulting aqueous solubility of CMN (*Y*_1_), as shown in Figure 5a. An increase in the concentration of SMP from 500 to 1,000 mg significantly enhanced the solubility of CMN from 0.34 to 0.76 mg/ml. This revealed that the carrier was effectively responsible for the aqueous solubility of the drug. On the other hand, the aqueous solubility of CMN slightly decreased from 70 to 40°C. The SR showed that a decrease in rpm (1,000 to 500) enhanced the aqueous solubility of CMN, although this effect was not prominent (Baig et al., 2016).

#### 3.6.2 Rel_5min_

The following equation (Equation 7) represents the polynomial model for the response (release at 5 min; *Y*_2_):

(7) $$\begin{array}{cc} Y_{2}= & +55.13+1.08*X_{1}‐0.47*X_{2}‐0.21*X_{3}+0.195*X_{1}*X_{2}‐0.95*X_{1}*X_{3}+0.13*X_{2}*X_{3}\\ & ‐4.11*X_{1}^{2}‐2.97*X_{2}^{2}‐0.89*X_{3}^{2}. \end{array}$$

Of all of the independent variables, CC was observed to have a significantly lower positive result on the release at 5 min as was manifest in the narrow positive value for its coefficient. The negative values for the SR and Temp showed that the release decreased at a higher rate of stirring and temperature.

The negative effect of increasing the stirring rate was found to be greater than the effect of increasing the Temp. The interaction between the independent variables was also found to be significant. Overall, the model was typical (*p* < .0001; *F*‐value = 102.46), while the lack of fit was not significant (*p* = .67; *F*‐value = 0.6115). The predicted value (.9517) and adj.*R* ^2^ (.9828) values are important parameters (Chaudhary et al., 2011). The CC significantly affected the release of CMN from SD at the end of 5 min: If the CC was too small, release from *SD* could not be achieved at the desired level; when CC was 1,000 mg, the release was up to 50%−55% (Figure 5b). Therefore, a suitable CC was selected for the release of CMN from SD. These results indicated that the release rate of CMN increased rapidly by increasing the SR and decreasing the temperature; these effects were significant for CC of 500 and 1,000 mg, and the center point of SR and Temp revealed that the release of CMN significantly increased as the temperature decreased, which could be due to complete amorphization of CMN.

The optimized formulation (P18) validated for the response variables (Sol_aq_ and Rel_5min_) was set at a maximum range, and the optimized preparation produced by the design noted as 850 mg of SMP, 750 rpm SR, and 40°C Temp. Accordingly, CMN‐SMP SD was prepared by the solvent evaporation method with these specifications. The experimental values of Sol_aq_ and Rel_5min_ (0.646 mg/ml and 54.94%, respectively) were compared with the predicted values (0.632 mg/ml and 55.71%), and the prediction error was found to be 1.02 and 0.98%, respectively. The negligible errors confirmed that the established model was sound, and the results of the predicted responses were in accordance with the investigational data. In conclusion, as a result of the BBD, a CMN‐SMP (1:8.5) inclusion complex with superior physicochemical properties was prepared. Subsequent experiments were carried out with this complex.

### 3.7 DLS

**FIGURE 6:**
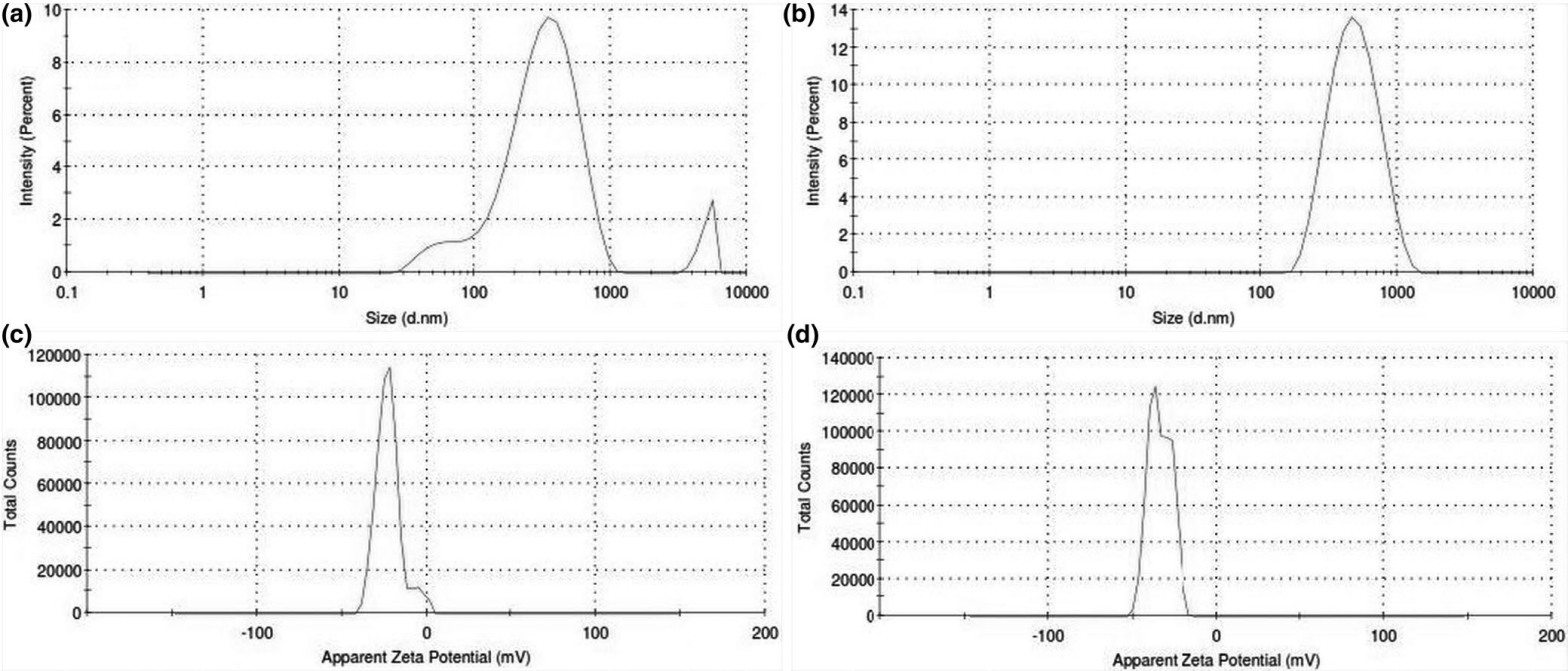
Hydrodynamic PS (a and b) and ZP (c and d) of PM (1:6) and SD (1:5) complex of CMN‐SMP

The hydrodynamic particle size of the PM was 383.4 nm and the PDI was 0.497 (Figure 6). This confirmed the bimodal size distribution, from which it was evident that the PM solution consisted of at least two constituents (Kumar et al., 2016). The hydrodynamic diameter of SD was 275.3 nm with a PDI of 0.239 (Figure 6b). The low PDI indicated the homogeneity of particle size distribution for the SD complex with CMN.

ZP is a significant parameter for understanding the state of the particle surface charge and stability (long‐term storage) of the products (Pooja et al., 2015). The ZP of the CMN‐PM and CMN‐SD complexes was found to be −23.2 and −33.9 mV, respectively (Figure 6c and d), which indicated that the powdered SD was molecularly dispersed with CMN to give a superior inclusion complex than the PM.

### 3.8 SEM

**FIGURE 7:**
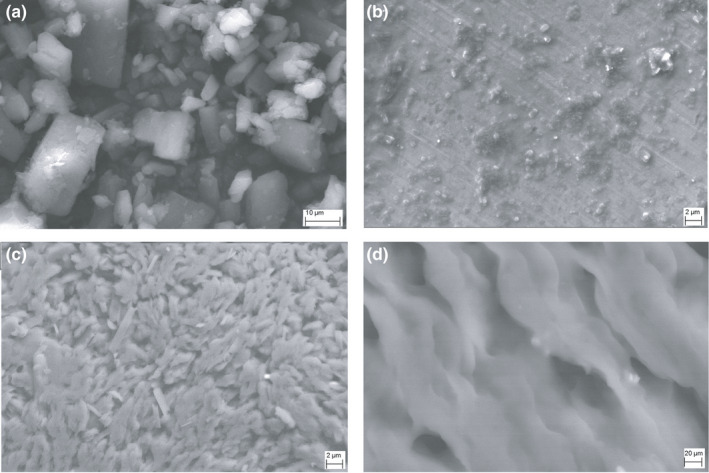
*SEM* image of (a) pure CMN, (b) SMP, (c) CMN‐SMP (PM), and (d) CMN‐SMP (SD)

Figure 7 shows the surface morphologies of (a) pure CMN, (b) pure SMP, (C) the PM, and (d) the SD. The pure CMN appeared as a characteristic prism‐surfaced crystalline structure with average particle size of 15 µm, and the SMP had a smooth surface with average particle size of 25 µm. The topological changes perceived in the SEM image of the PM complex seem to be the result of partial conversion of crystalline CMN to an amorphous structure. This change indicated the commencement of its partial inclusion complexation.

In contrast, in the SD image (Figure 7d) the drug surface appeared to be more porous, uniform and homogeneously dispersed. The morphology of both the CMN and SMP had disappeared at the molecular level, revealing their complete conversion into the molecular level inclusion form.

### 3.9 *In vitro* cytotoxicity of SMP inclusion complex

**FIGURE 8:**
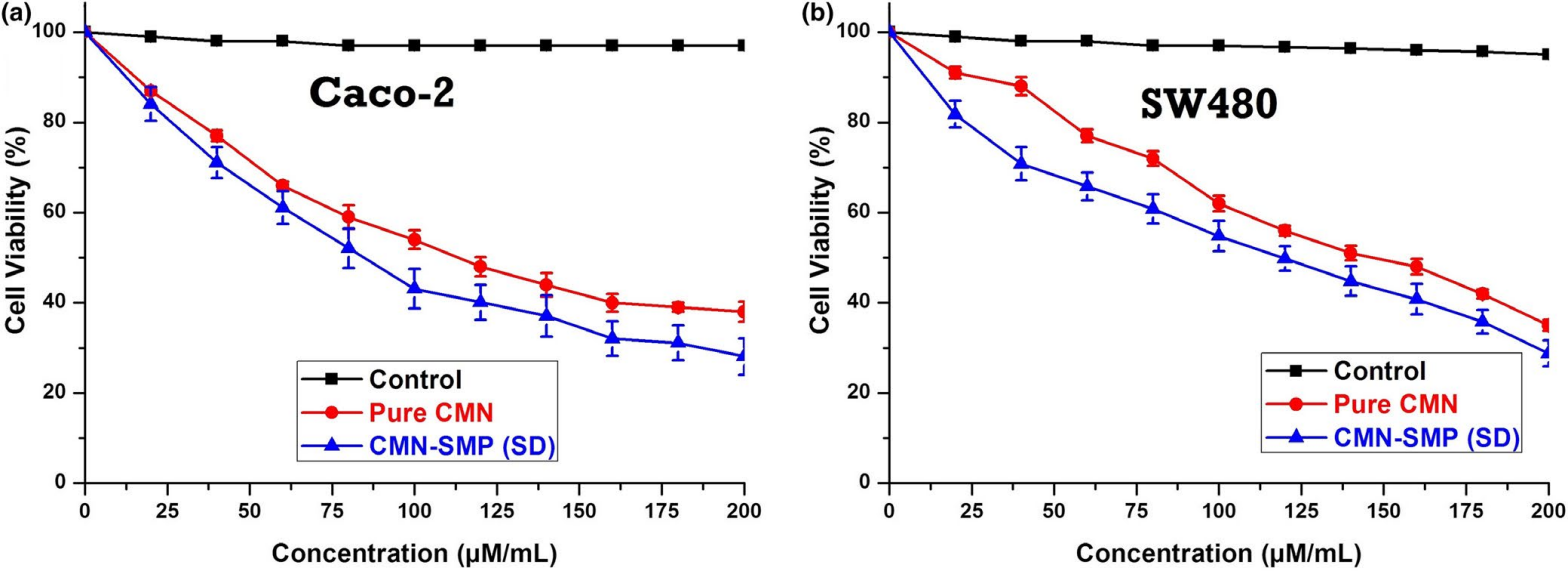
The in vitro cytotoxic effect of CMN and CMN‐*SD* on (a) Caco‐2 and (b) SW480 cells lines (Mean ± *SD*, *n* = 3)

The cytotoxic outcomes of the MTT reduction assay of pure CMN and optimized CMN‐SMP inclusion complex on Caco‐2 (Figure 8a) and SW480 and cells lines are shown in Figure 8b. The IC_50_ values for the complexes were calculated as being around 73 and 89 µM/ml for SW480 and Caco‐2, respectively, whereas pure CMN was 116 and 132 µM/ml (Sun et al., 2013).

The outcomes of the MTT assay showed that the inclusion complex exhibited better cytotoxicity than pure CMN. This could be due to variations in cellular uptake leading to the improved effect of the complex (Manju & Sreenivasan, 2012). The effects of this assay indicated that the complex could transport the drug to Caco‐2 and SW480 cells using endocytic process by active targeting combined with better cytotoxicity and aqueous solubility of CMN. The cell viability gradually declined with increasing concentration of C‐SD and pure CMN, while SD (soluble CMN) showed a significantly better cytotoxic effect than pure CMN.

### 3.10 Cell Death

#### 3.10.1 AO/EB staining

**FIGURE 9:**
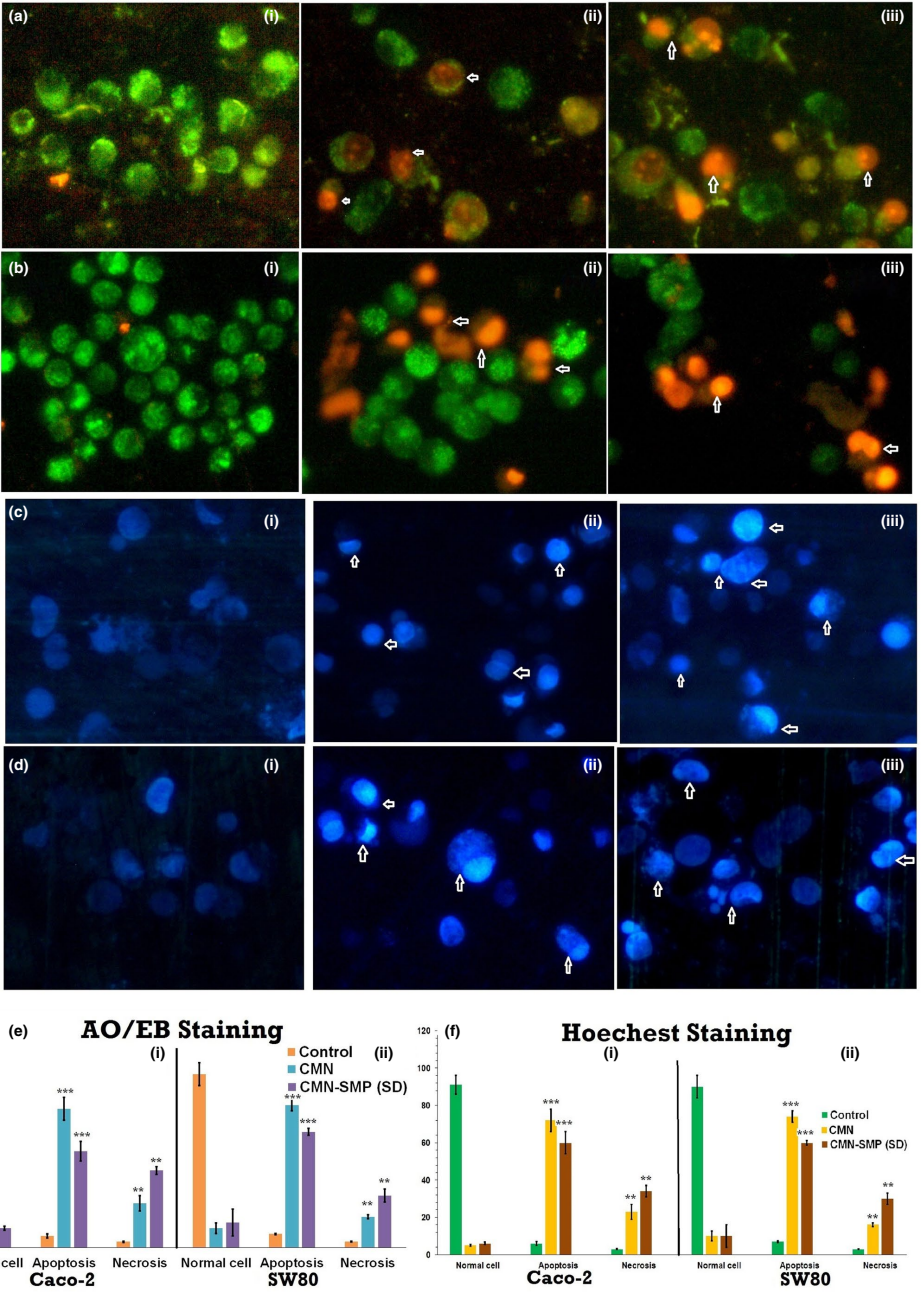
Apoptotic morphology of (a, b) AO/EB and (c and d) Hoechst staining pictured using fluorescent microscope with (a, c) Caco‐2 and (b and d) SW480 cells; (i) control; (ii) pure CMN; and (iii) CMN‐SMP inclusion complex and percentage of normal, apoptotic, and necrotic cells (e and f) at 24‐hr treatment (***p < .001 calculated by the one‐way ANOVA test)

The cytotoxic effect produced by the inclusion complex on Caco‐2 and SW480 cells was shown in terms of cell viability or control cells. Unbroken chromatin with an undamaged cell membrane appeared as a bright green color, indicating that the cells did not experience apoptotic changes. The complex SD treatment produced highly effective apoptosis, increasing both necrosis and nonviable cells, compared to treatment with pure CMN. Types of morphological changes such as cytological and geologies of the chromatin changes (Abel & Sarah, 2018) during apoptosis were measured and the cells were classified as follows. (a) Viable cells: The cells had a well‐organized structure and uniform chromatin with a green fluorescing nucleus (Figure 9a and b; i). (b) Early apoptotic cells: Fragmentation of DNA had just begun, and there was intact cell structure and a build‐up of chromatin with green fluorescing nuclei (Figure 9a and b; ii and iii); (c) late apoptotic cells: DNA and chromatins were cleaved/condensed or damaged with orange to red fluorescing nuclei (Figure 9a and b; ii and iii); and (d) necrotic cells: The cell membrane was a swollen or substantial structure, and DNA and chromatin fragmentations were absent, with orange to red fluorescing nucleus (Figure 9a and b; ii and iii). The outcomes of this study suggested that the SD complex treatment caused cell death mainly through apoptosis and a small amount necrosis (Figure 9e); this was true for both Caco‐2 and SW480 and was exhibited more potential cytotoxic effect than with pure CMN treatment.

#### 3.10.2 Hoechst staining

Cytological changes of the SW480 and Caco‐2 cells were detected after treatment with the IC_50_ concentrations of the complexes for 24 hr (Vignesh et al., 2014). A manual count of normal and abnormal cells was performed using Hoechst 33258 staining of the Sw480 and Caco‐2 cells, shown in Figure 9c,d (Ramakrishnan et al., 2011).

This method showed abnormalities in the cell cytology, with specific reference to the nucleus core and cytoplasm at the primary level to identify apoptosis (Figure 9c,d; ii and iii). Both late and early apoptotic cells appeared as cell shrinkage with an intact membrane structure, chromatin build‐up with discontinuity of DNA (Dhivya et al., 2015). A small number of necrotic cells were also observed (Figure 9c,d; ii and iii). The percentage of viable, nonviable, apoptotic, and necrotic cells were estimated (Figure 9f).

### 3.11 DNA fragmentation

**FIGURE 10:**
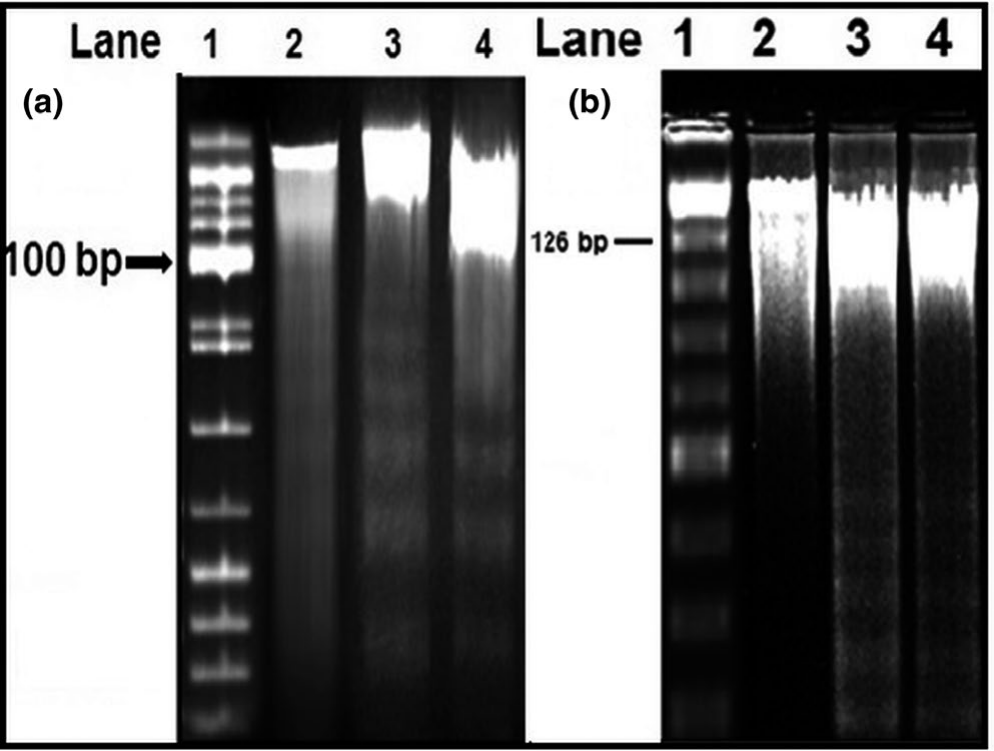
Gel electrophoresis of DNA of treated Caco‐2 and SW480 cells; Lane 1:100 and 126 bp ladder, Lane 2: Control, Lane 3: Pure CMN, Lane 4: CMN‐SMP (SD)

To trigger apoptosis in CMN‐treated Caco‐2 and SW480 cells, a DNA fragmentation assay was performed using the agarose gel electrophoresis method. This reveals “fragment or ladder” pattern of DNA and ∼100 and ∼126 bp intervals (Lane 1). Lanes were loaded with equimolar concentrations of DNA extracted from cells of control, pure CMN‐treated, and SD complex‐treated Caco‐2 (Figure 10a) and SW480 (Figure 10b) cells. Lane 2 (control) showed a DNA band with an unbroken pattern. CMN‐treated lanes (3 and 4) had a smeared pattern owing to DNA fragmentation occurring. Greater smearing or laddering of DNA was observed in the lane treated with SD complex due greater DNA fragmentation occurring; this indicated that apoptosis occurred by the action of nuclear enzyme oligonucleosomes (Vignesh et al., 2014). An in‐depth visualization displayed the outstanding outcome on the initiation of apoptosis as evidenced by the presence of DNA ladders in the samples treated with inclusion complex, exhibiting more active cell death compared with pure CMN (more cleavage of the DNA “ladder”).

### 3.12 Cell cycle analysis

**FIGURE 11:**
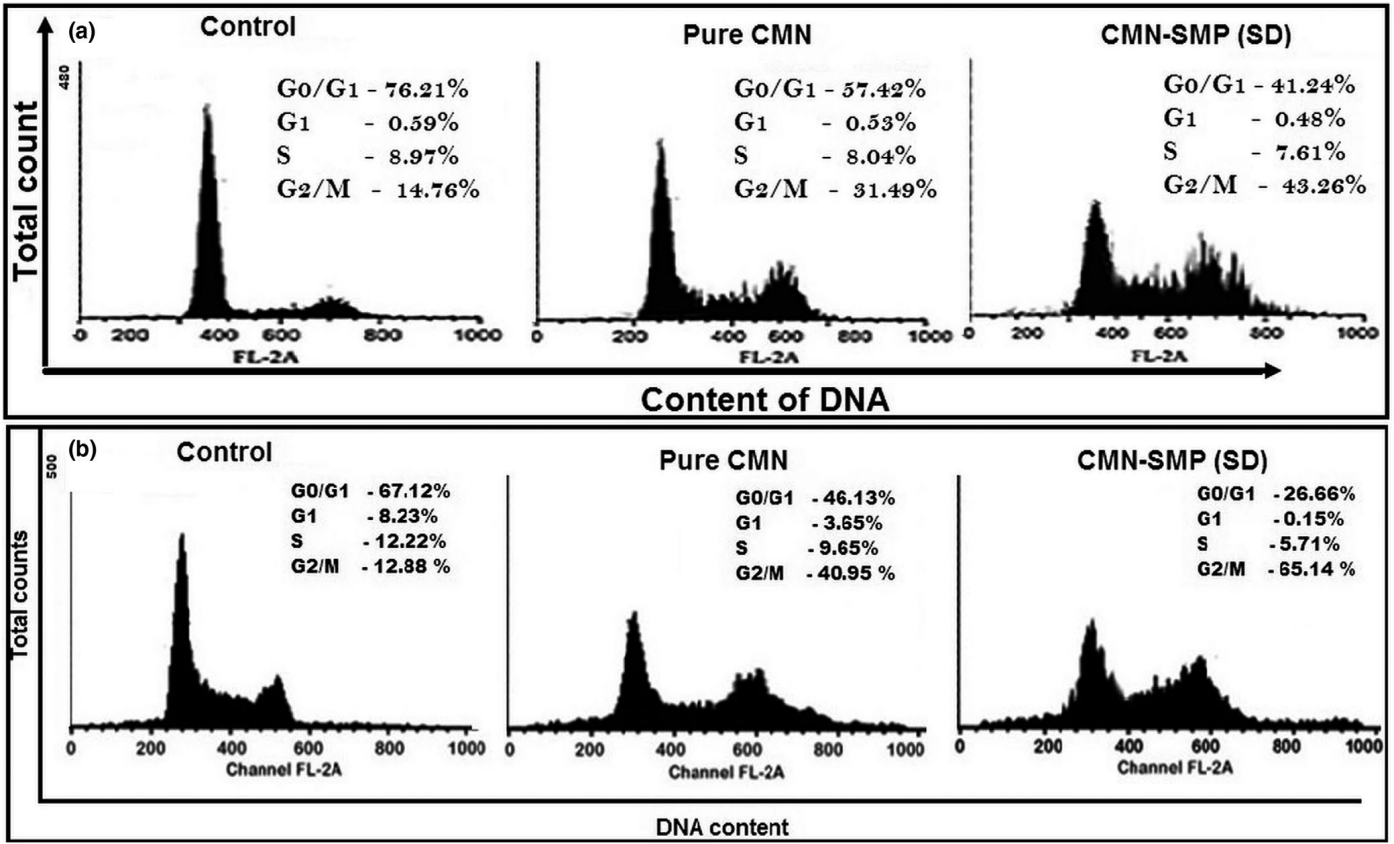
Effects of CMN‐SMP (SD) on Caco‐2 and SW480 cell cycle analysis; the nonsmall colon cancer cells were cultured for 24 hr

Propidium iodide staining was used to enable morphological investigation by flow cytometry. After 24 hr of treatment, the Caco‐2 and SW480 cells were analyzed for the stage of cell cycle arrest and initiation of apoptosis. At G0/M phase, only 14.76 (Figure 11a) and 12.88% (Figure 11b) of the cell population was observed at control, whereas 31.49% and 40.95% and 43.26% and 65.14% of the cells treated with pure CMN and the SD complex, respectively, were at G2/M phase. While increasing the cell population at G2/M phase, the cell population at G1 and S phases subsequently decreased (0.48% and 7.61% for Caco‐2 and 0.15% and 5.71% for SW480 cells) after treatment with pure CMN and the SD complex. In conclusion, the CMN inclusion complex effectively blocked the colorectal adenocarcinoma cells at the G2/M phase: The cytotoxicity of CMN killed the cancer cells by triggering cell death via numerous caspase‐mediated paths (Oberhammer et al., 1993), which activated the apoptotic pathway subsequently treated with SMP inclusion complex.

## 4 CONCLUSION

In conclusion, this study determined that the ideal stoichiometric ratio of CMN with carrier to enhance its solubility was 1:1. This complex was found to be more effective against colorectal cancer (CRC) than pure CMN. The solubility and dissolution properties of CMN complexes with carriers PVP, MNT, and SMP were determined. SMP formed an ideal complex with CMN in the molecular level inclusion, which may have responsible for enhancing the solubility of CMN. Phase solubility studies revealed this to be the most soluble complex. The CMN‐SMP inclusion complex displayed significantly enhanced anticancer activity against Caco‐2 and SW480 cells, with anticancer potency derived mostly from the induction of cellular apoptosis of tumor cells. Flow cytometry analysis confirmed that cell cycle arrest was at G2/M phase, and DNA fragmentation analysis indicated that the complex induced significant DNA damage during apoptosis. The profound efficiency of the optimized CMN‐SMP inclusion complex indicated its suitability for the production of soluble products and exploration in the treatment of CRC.

## CONFLICT OF INTEREST

The authors declare that they have no conflict of interests on this research work.

## AUTHORS CONTRIBUTION

Muthu Mohamed Jamal Moideen involved in conceptualization, formal analysis, investigation, methodology, software, validation, and writing‐original draft. Ali Alqahtani involved in project adminstration, resources, review, and editing. Krishnaraju Venkatesan acquired funding, wrote—reviewed and edited. Fazil Ahmad involved in data curation and writing—review and editing. Kalpana Krisharaju involved in resources and writing—review and editing. Mohammed Gayasuddin involved in resources, funding acquisition. Rasheed Ahemad Shaik involved in resources, funding acquisition. Khalid Mohamad Morsy Ibraheem involved in software, funding acquisition. Mohamed EL‐dosoky Mohamed Salama in resources, funding acquisition. Sally Yussef Abed involved in software, resources, funding acquisition.
